# Incidence and survival for cancer in children and young adults in the North of England, 1968–1995: a report from the Northern Region Young Persons’ Malignant Disease Registry

**DOI:** 10.1054/bjoc.2000.1313

**Published:** 2000-07-03

**Authors:** S J Cotterill, L Parker, A J Malcolm, M Reid, L More, A W Craft

**Affiliations:** Department of Child Health, University of Newcastle upon Tyne, Queen Victoria Road, Newcastle, NE1 4LP, UK

**Keywords:** oncology, childhood, adolescents, young adults, incidence, survival

## Abstract

The Northern Region Young Persons’ Malignant Disease Registry records information on young people under 25 years old diagnosed with cancer in the Northern Region of England. Incidence and survival rates were calculated for children and young adults diagnosed with cancer between 1968 and 1995. There were 2099 (M:F 1.28:1) children (age 0–14 years) and 2217 (M:F 1.23:1) young adults (15–24 years) diagnosed with a first cancer between 1968 and 1995. The age-standardized rate (ASR) for childhood cancer was 121 per million 0 to 14 year-olds per year. For young adults the ASR was 175 per million 15 to 24 year-olds, per year. Incidence of childhood cancer increased over time at a rate of 12 extra cases per million children, per decade (*P*< 0.001). In young adults incidence rates increased by 16 extra cases per million 15 to 24 year-olds, per decade (*P*< 0.001). For childhood cancer 5-year survival was 42% for those diagnosed 1968–1977, 57% for 1978–1987 and 71% (95% CI 67–75) for 1988–1995. Survival for young adults over the three periods was 45%, 62% and 73% (95% CI 70–78) respectively. The cumulative risk of developing cancer before the age of 25 is 1 in 285. Over the 28-year period there were significant improvements in survival and modest increases in incidence in both children and young adults. © 2000 Cancer Research Campaign


*British Journal of Cancer* (2000) **83**(3), 397–403
© 2000 Cancer Research Campaign
doi: 10.1054/ bjoc.2000.1313, available online at http://www.idealibrary.com on IDEAL®

### Incidence and survival for cancer in children and young adults in the North of England, 1968–1995: a report from the Northern Region Young Persons' Malignant Disease Registry

**SJ Cotterill, L Parker, AJ Malcolm, M Reid, L More and AW Craft**

Department of Child Health, University of Newcastle upon Tyne, Queen Victoria Road, Newcastle NE1 4LP, UK

**Summary** The Northern Region Young Persons' Malignant Disease Registry records information on young people under 25 years old diagnosed with cancer in the Northern Region of England. Incidence and survival rates were calculated for children and young adults diagnosed with cancer between 1968 and 1995. There were 2099 (M:F 1.28:1) children (age 0–14 years) and 2217 (M:F 1.23:1) young adults (15–24 years) diagnosed with a first cancer between 1968 and 1995. The age-standardized rate (ASR) for childhood cancer was 121 per million 0 to 14 year-olds per year. For young adults the ASR was 175 per million 15 to 24 year-olds, per year. Incidence of childhood cancer increased over time at a rate of 12 extra cases per million children, per decade ($P < 0.001$). In young adults incidence rates increased by 16 extra cases per million 15 to 24 year-olds, per decade ($P < 0.001$). For childhood cancer 5-year survival was 42% for those diagnosed 1968–1977, 57% for 1978–1987 and 71% (95% CI 67–75) for 1988–1995. Survival for young adults over the three periods was 45%, 62% and 73% (95% CI 70–78) respectively. The cumulative risk of developing cancer before the age of 25 is 1 in 285. Over the 28-year period there were significant improvements in survival and modest increases in incidence in both children and young adults. © 2000 Cancer Research Campaign

**Keywords:** oncology; childhood; adolescents; young adults; incidence; survival

The Northern Region Young Persons' Malignant Disease Registry (NRYPMDR) established in 1968 (Craft et al, 1987), was one of the first population-based childhood cancer registries in the UK, the earliest being the Manchester Children's Tumour Registry (Birch, 1988). It is the only specialist registry in the UK currently recording cancer in young adults aged under 25 years. This study aimed to document incidence and survival from cancer in children (aged 0–14 years) and young adults (aged 15–24 years) diagnosed in the North of England during the period 1968 to 1995, and to examine changes in incidence rates and survival over time.

### PATIENTS AND METHODS

### The Northern Region Young Persons' Malignant Disease Registry

The NRYPMDR was established in 1968 and covers the Northern Region of England as defined by 1972 Boundary Commission (Compten, 1972), excluding the district of Barrow-in-Furness. The study region (Figure 1) has a population of 3.1 million, with approximately 37 000 live births per year and covers an area of 15 337 km$^2$ with a mixture of urban and rural areas. Population density varies widely; Northumberland and Cumbria are the two most sparsely populated counties in England, while Tyne & Wear is one of the most densely populated (HMSO, 1996). The population is predominantly Caucasian and ethnic minorities account for under 2%. Data on malignancies and benign central nervous systems (CNS) tumours in children resident in the region at time of diagnosis have been collected prospectively since 1968 and incidence and survival up to 1982 have been reported previously (Craft et al, 1987). Cancers in the 15 to 24 year age group for the period 1968 to 1985 were identified retrospectively in 1985 (Craft et al, 1993) and have been collected prospectively since then.

Cases are identified from multiple sources: consultants in the region notify the registry of any malignancies in children and, young adults. Data are periodically cross-checked with regional and national cancer registries; death certificates and hospital admissions are scrutinized regularly. The registry liaises with the National Children's Tumour Registry in Oxford and the United Kingdom Children's Cancer Study Group to ensure information is as accurate and complete as possible. Ascertainment is believed to be at least 98% complete. Responsible clinicians and general practitioners are contacted at regular intervals (approximately 6-monthly) to determine patients' current vital status. The registry does not record cervical intra-epithelial neoplasia.

Data collected by the NRYPMDR include demographic details (such as age, gender, residential address) and details of diagnosis and treatment. A copy of the birth certificate is obtained by the registry and cases undergo central diagnostic and pathological review. Tumours are coded for morphology and primary site using the International Classification of Diseases for Oncology (ICDO-2) (WHO, 1990). Cases are grouped according to a modified

*Received 26 January 2000*
*Revised 28 February 2000*
*Accepted 6 March 2000*

*Correspondence to:* SJ Cotterill

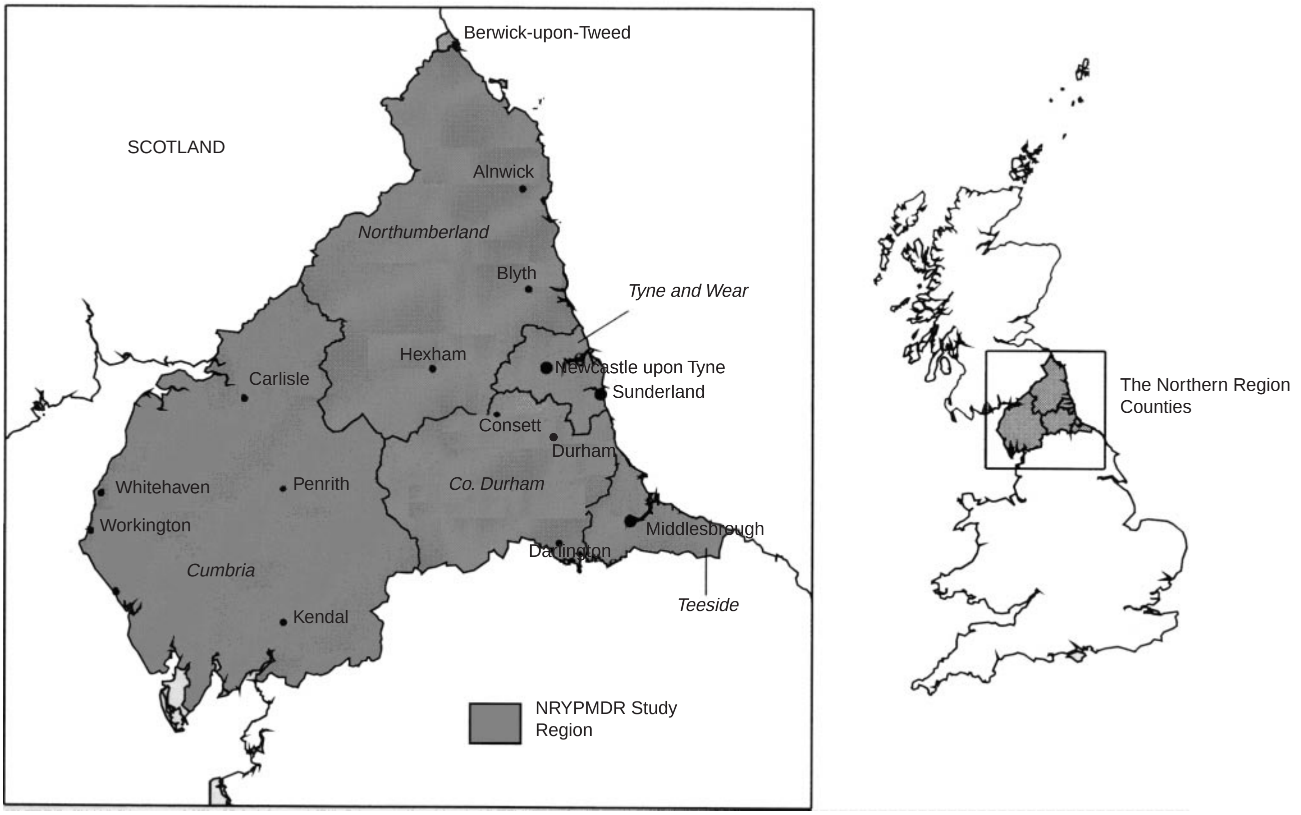

**Figure 1** The study region covered by the Northern Region Young Persons' Malignant Disease Registry version of the International Classification of Childhood Cancer (ICCC) (Kramarova et al, 1996). The modifications are as follows:

1. Germ cell and gonadal tumours (group Xc) is subdivided into seminoma (M9060–9064), testicular endodermal tumours (M9070–9079), testicular teratoma (M9080–9102), and germ cell tumours of the ovary.
2. Other carcinomas (group Xf) are subdivided into lung (C34.0–34.9), colorectal (C18.0–20.9), breast (C50.0–50.9), cervix (C53.8–53.9), and other sites
3. Langerhans cell histiocytosis (LCH) is reported as a separate group and not included in further analysis.

Data verification programs are applied to check the validity of morphology, primary site and gender combinations. Second and subsequent primary cancers (51), tumours of benign or uncertain behavior (56) and carcinomas in situ (8) were excluded from the analyses.

### Data analysis

Incidence rates were calculated based on mid-year population estimates for the study region obtained from the National Health Service Executive, Durham. Adjustments for the population of Barrow-in-Furness were made based on census estimates. Age-standardized rates (ASR) were calculated based on the standard world population (Smith, 1992). Cumulative rates and cumulative risks were also calculated (Day, 1992). Trends for changes in annual ASR were analysed by single linear regression. Survival rates were calculated using Kaplan–Meier estimation (Kaplan and Meier, 1958).

### RESULTS

### Incidence

There were 4316 patients aged under 25 years diagnosed with a first cancer in the study region between 1968 and 1995; 2099 (M:F 1.28:1) children (age < 15 years) and 2217 (M:F 1.23:1) young adults (age 15–24 years). In addition there were 46 cases of Langerhans cell histiocytosis (LCH). Number of cases and age-specific incidence rates by disease type for the period 1968–1995 are shown in Table 1. Of two cases classed as hepatoblastoma in the 20 to 24 year group only one with carcinoid syndrome had central review of pathology; it was compatible with a primary hepatoma though metastases from a distant site could not be completely ruled out. The 'other specified sarcomas' (group IXd) composed of synovial sarcoma (25), leiomyosarcoma (11), soft tissue Ewing's/pPNET (10), hemangiopericytoma (4), myxoid liposarcoma (3), hemangiosarcoma (3), myxosarcoma (2), pleomorphic liposarcoma (2), clear cell sarcoma (2), alveolar soft part sarcoma (2), other unique sarcomas (12). In the non-melanoma skin carcinoma (Group XIe) basal cell carcinoma was the most frequent type ($n$ = 40) with squamous cell carcinoma accounting for six cases. The principle sites of the 'other and unspecified carcinomas' (group XIf5) were carcinoma of unknown primary (ICDO C80.9 $n$ = 18), urethra and other urinary organs (C68 $n$ = 17), salivary glands (C8 $n$ = 10), small intestine (C17 $n$ = 7) and mediastinum (C38 $n$ = 7). The remaining 53 carcinomas were from a heterogeneous range of sites each with fewer than five cases.

© 2000 Cancer Research Campaign

**Table 1** Number of cases and incidence rates by disease type for the period 1968–1995 per million per year

| | | Number of cases Age-group (years) | | | | | | Incidence rates (per million; per year) Age-group (years) | | | | | | | ASR | ASR | Gender ratio |
|---|---|---|---|---|---|---|---|---|---|---|---|---|---|---|---|---|---|
| **Diagnostic Group** | | **0–** | **1–4** | **5–9** | **10–14** | **15–19** | **20–24** | **0–** | **1–4** | **5–9** | **10–14** | **15–19** | **20–24** | **CUM** | **0–14** | **15–24** | **M:F** |
| I | Leukaemia | 32 | 324 | 190 | 130 | 150 | 103 | 28.3 | 69.4 | 30.9 | 20.5 | 24.0 | 16.4 | 765 | 39.6 | 20.4 | 1.38 |
| | a Lymphoid leukaemia | 21 | 292 | 161 | 94 | 82 | 43 | 18.6 | 62.6 | 26.2 | 14.8 | 13.1 | 6.8 | 574 | 33.6 | 10.2 | 1.36 |
| | b Acute non-lymphocytic | 9 | 28 | 26 | 29 | 54 | 41 | 8.0 | 6.0 | 4.2 | 4.6 | 8.6 | 6.5 | 152 | 5.2 | 7.6 | 1.37 |
| | c Chronic myeloid | 1 | 2 | 2 | 5 | 9 | 15 | 0.9 | 0.4 | 0.3 | 0.8 | 1.4 | 2.4 | 27 | 0.5 | 1.9 | 2.09 |
| | d Other specified | 0 | 1 | 1 | 1 | 2 | 0 | 0.0 | 0.2 | 0.2 | 0.2 | 0.3 | 0.0 | 4 | 0.2 | 0.2 | 0.25 |
| | e Unspecified | 1 | 1 | 0 | 1 | 3 | 4 | 0.9 | 0.2 | 0.0 | 0.2 | 0.5 | 0.6 | 8 | 0.2 | 0.6 | 2.33 |
| II | Lymphomas | 2 | 31 | 73 | 97 | 243 | 332 | 1.8 | 6.6 | 11.9 | 15.3 | 38.9 | 52.8 | 623 | 10.5 | 45.4 | 1.87 |
| | a Hodgkin's disease | 0 | 4 | 27 | 54 | 176 | 258 | 0.0 | 0.9 | 4.4 | 8.5 | 28.2 | 41.1 | 414 | 4.2 | 34.2 | 1.78 |
| | b Non-Hodgkin's | 2 | 26 | 42 | 41 | 64 | 63 | 1.8 | 5.6 | 6.8 | 6.5 | 10.2 | 10.0 | 192 | 5.9 | 10.1 | 2.05 |
| | c Burkitt's lymphoma | 0 | 0 | 2 | 1 | 1 | 3 | 0.0 | 0.0 | 0.3 | 0.2 | 0.2 | 0.5 | 6 | 0.2 | 0.3 | 2.50 |
| | d Misc. lymphoreticular | 0 | 0 | 1 | 1 | 0 | 1 | 0.0 | 0.0 | 0.2 | 0.2 | 0.0 | 0.2 | 2 | 0.1 | 0.1 | 0.67 |
| | e Unspecified lymphomas | 0 | 1 | 1 | 0 | 2 | 7 | 0.0 | 0.2 | 0.2 | 0.0 | 0.3 | 1.1 | 9 | 0.1 | 0.7 | 4.50 |
| III | CNS tumours | 42 | 148 | 168 | 137 | 141 | 176 | 37.2 | 31.7 | 27.3 | 21.6 | 22.6 | 28.0 | 662 | 27.8 | 25.1 | 1.29 |
| | a Ependymoma | 14 | 16 | 15 | 8 | 13 | 9 | 12.4 | 3.4 | 2.4 | 1.3 | 2.1 | 1.4 | 62 | 3.2 | 1.8 | 2.75 |
| | b Astrocytoma | 6 | 50 | 52 | 52 | 47 | 52 | 5.3 | 10.7 | 8.5 | 8.2 | 7.5 | 8.3 | 210 | 8.8 | 7.9 | 1.25 |
| | c Pnet | 7 | 37 | 26 | 19 | 6 | 11 | 6.2 | 7.9 | 4.2 | 3.0 | 1.0 | 1.8 | 88 | 5.2 | 1.3 | 1.74 |
| | d Other gliomas | 5 | 16 | 18 | 11 | 21 | 33 | 4.4 | 3.4 | 2.9 | 1.7 | 3.4 | 5.3 | 85 | 2.9 | 4.3 | 1.21 |
| | e Other specified | 4 | 13 | 13 | 30 | 38 | 48 | 3.5 | 2.8 | 2.1 | 4.7 | 6.1 | 7.6 | 118 | 3.2 | 6.8 | 1.26 |
| | f Unspecified | 6 | 16 | 44 | 17 | 16 | 23 | 5.3 | 3.4 | 7.2 | 2.7 | 2.6 | 3.7 | 99 | 4.6 | 3.1 | 0.74 |
| IV | Sympathetic nervous system | 35 | 79 | 21 | 8 | 5 | 5 | 31.0 | 16.9 | 3.4 | 1.3 | 0.8 | 0.8 | 130 | 9.1 | 0.8 | 1.28 |
| | a Neuroblastoma | 35 | 74 | 20 | 6 | 5 | 2 | 31.0 | 15.9 | 3.3 | 0.9 | 0.8 | 0.3 | 121 | 8.6 | 0.6 | 1.25 |
| | b Other | 0 | 5 | 1 | 2 | 0 | 3 | 0.0 | 1.1 | 0.2 | 0.3 | 0.0 | 0.5 | 9 | 0.5 | 0.2 | 1.75 |
| V | Retinoblastoma | 31 | 44 | 0 | 0 | 0 | 0 | 27.5 | 9.4 | 0.0 | 0.0 | 0.0 | 0.0 | 65 | 5.0 | 0.0 | 0.67 |
| VI | Renal tumours | 15 | 80 | 20 | 4 | 3 | 9 | 13.3 | 17.1 | 3.3 | 0.6 | 0.5 | 1.4 | 111 | 7.6 | 0.9 | 0.87 |
| | a Wilms' & clear cell sarcoma | 15 | 80 | 20 | 3 | 0 | 1 | 13.3 | 17.1 | 3.3 | 0.5 | 0.0 | 0.2 | 101 | 7.5 | 0.1 | 0.86 |
| | b Renal Ccarcinoma | 0 | 0 | 0 | 1 | 3 | 8 | 0.0 | 0.0 | 0.0 | 0.2 | 0.5 | 1.3 | 10 | 0.0 | 0.9 | 1.00 |
| VII | Hepatic tumours | 5 | 7 | 1 | 1 | 3 | 4 | 4.4 | 1.5 | 0.2 | 0.2 | 0.5 | 0.6 | 18 | 0.9 | 0.6 | 0.62 |
| | a Hepatoblastoma | 5 | 5 | 0 | 1 | 0 | 2 | 4.4 | 1.1 | 0.0 | 0.2 | 0.0 | 0.3 | 11 | 0.7 | 0.1 | 0.44 |
| | b Hepatic carcinoma | 0 | 2 | 1 | 0 | 3 | 2 | 0.0 | 0.4 | 0.2 | 0.0 | 0.5 | 0.3 | 7 | 0.2 | 0.4 | 1.00 |
| VIII | Bone tumours | 0 | 7 | 19 | 74 | 92 | 43 | 0.0 | 1.5 | 3.1 | 11.7 | 14.7 | 6.8 | 188 | 4.9 | 11.0 | 1.59 |
| | a Osteosarcoma | 0 | 4 | 10 | 45 | 53 | 19 | 0.0 | 0.9 | 1.6 | 7.1 | 8.5 | 3.0 | 105 | 2.9 | 5.9 | 1.43 |
| | b Chondrosarcoma | 0 | 0 | 1 | 3 | 4 | 7 | 0.0 | 0.0 | 0.2 | 0.5 | 0.6 | 1.1 | 12 | 0.2 | 0.9 | 2.00 |
| | c Ewing's sarcoma | 0 | 3 | 8 | 25 | 30 | 15 | 0.0 | 0.6 | 1.3 | 3.9 | 4.8 | 2.4 | 65 | 1.8 | 3.7 | 1.89 |
| | d Other specified | 0 | 0 | 0 | 0 | 4 | 2 | 0.0 | 0.0 | 0.0 | 0.0 | 0.6 | 0.3 | 5 | 0.0 | 0.5 | 1.40 |
| | e Unspecified | 0 | 0 | 0 | 1 | 1 | 0 | 0.0 | 0.0 | 0.0 | 0.2 | 0.2 | 0.0 | 2 | 0.0 | 0.1 | 1.00 |
| IX | Soft tissue sarcomas | 9 | 48 | 45 | 33 | 56 | 51 | 8.0 | 10.3 | 7.3 | 5.2 | 9.0 | 8.1 | 197 | 7.7 | 8.6 | 1.14 |
| | a Rhabdomyosarcoma | 2 | 40 | 31 | 11 | 21 | 5 | 1.8 | 8.6 | 5.0 | 1.7 | 3.4 | 0.8 | 91 | 4.9 | 2.2 | 1.29 |
| | b Fibrosarcoma, neurofibrosarcoma | 6 | 3 | 6 | 5 | 12 | 16 | 5.3 | 0.6 | 1.0 | 0.8 | 1.9 | 2.5 | 39 | 1.2 | 2.2 | 0.85 |
| | d Other specified | 1 | 5 | 8 | 15 | 20 | 27 | 0.9 | 1.1 | 1.3 | 2.4 | 3.2 | 4.3 | 61 | 1.5 | 3.7 | 1.16 |
| | e Unspecified | 0 | 0 | 0 | 2 | 3 | 3 | 0.0 | 0.0 | 0.0 | 0.3 | 0.5 | 0.5 | 6 | 0.1 | 0.5 | 1.67 |
| X | Germ cell/gonadal | 14 | 31 | 7 | 18 | 80 | 227 | 12.4 | 6.6 | 1.1 | 2.8 | 12.8 | 36.1 | 303 | 4.2 | 23.8 | 1.83 |
| | a Intracranial germ cell | 1 | 1 | 3 | 8 | 4 | 4 | 0.9 | 0.2 | 0.5 | 1.3 | 0.6 | 0.6 | 17 | 0.7 | 0.6 | 1.10 |
| | b Other non-gonadal germ cell | 6 | 10 | 1 | 1 | 8 | 22 | 5.3 | 2.1 | 0.2 | 0.2 | 1.3 | 3.5 | 39 | 1.2 | 2.3 | 0.60 |
| | c1 Testes seminoma* | 0 | 0 | 0 | 0 | 2 | 33 | 0.0 | 0.0 | 0.0 | 0.0 | 0.6 | 10.4 | 55 | 0.0 | 5.2 | – |
| | c2 Testes endodermal* | 6 | 18 | 0 | 0 | 1 | 6 | 10.4 | 7.5 | 0.0 | 0.0 | 0.3 | 1.9 | 51 | 3.1 | 1.1 | – |
| | c3 Testes Teratoma* | 1 | 1 | 1 | 0 | 42 | 102 | 1.7 | 0.4 | 0.3 | 0.0 | 13.3 | 32.2 | 233 | 0.4 | 22.2 | – |
| | c4 Ovary germ cell* | 0 | 0 | 2 | 9 | 8 | 11 | 0.0 | 0.0 | 0.7 | 2.9 | 2.6 | 3.5 | 49 | 1.1 | 3.0 | – |
| | d Gonadal Ccarcinoma | 0 | 0 | 0 | 0 | 13 | 41 | 0.0 | 0.0 | 0.0 | 0.0 | 2.1 | 6.5 | 43 | 0.0 | 4.2 | 0.02 |
| | e Other and unspecified | 0 | 1 | 0 | 0 | 2 | 8 | 0.0 | 0.2 | 0.0 | 0.0 | 0.3 | 1.3 | 9 | 0.1 | 0.8 | 0.10 |
| XI | Carcinomas | 4 | 11 | 10 | 39 | 124 | 360 | 3.5 | 2.4 | 1.6 | 6.2 | 19.8 | 57.3 | 438 | 3.3 | 37.5 | 0.52 |
| | a Adrenocortical | 1 | 1 | 0 | 0 | 1 | 1 | 0.9 | 0.2 | 0.0 | 0.0 | 0.2 | 0.2 | 3 | 0.1 | 0.2 | 1.00 |
| | b Thyroid | 0 | 2 | 3 | 7 | 24 | 35 | 0.0 | 0.4 | 0.5 | 1.1 | 3.8 | 5.6 | 57 | 0.6 | 4.7 | 0.48 |
| | c Nasopharyngeal | 0 | 1 | 1 | 4 | 6 | 8 | 0.0 | 0.2 | 0.2 | 0.6 | 1.0 | 1.3 | 16 | 0.3 | 1.1 | 1.00 |
| | d Malignant melanoma | 2 | 3 | 1 | 10 | 41 | 89 | 1.8 | 0.6 | 0.2 | 1.6 | 6.6 | 14.2 | 117 | 0.8 | 10.1 | 0.49 |
| | e Skin carcinoma | 0 | 0 | 0 | 5 | 11 | 32 | 0.0 | 0.0 | 0.0 | 0.8 | 1.8 | 5.1 | 38 | 0.2 | 3.3 | 0.78 |
| | f1 Lung carcinoma | 0 | 0 | 1 | 1 | 6 | 10 | 0.0 | 0.0 | 0.2 | 0.2 | 1.0 | 1.6 | 14 | 0.1 | 1.3 | 2.00 |
| | f2 Breast carcinoma* | 0 | 0 | 0 | 0 | 1 | 33 | 0.0 | 0.0 | 0.0 | 0.0 | 0.3 | 10.6 | 55 | 0.0 | 5.2 | – |
| | f3 Colorectal carcinoma | 0 | 0 | 0 | 1 | 11 | 16 | 0.0 | 0.0 | 0.0 | 0.2 | 1.8 | 2.5 | 22 | 0.0 | 2.1 | 2.50 |
| | f4 Cervical carcinoma* | 0 | 0 | 0 | 0 | 1 | 66 | 0.0 | 0.0 | 0.0 | 0.0 | 0.3 | 21.2 | 107 | 0.0 | 10.1 | – |
| | f5 Other and unspecified | 1 | 4 | 4 | 11 | 22 | 70 | 0.9 | 0.9 | 0.7 | 1.7 | 3.5 | 11.1 | 90 | 1.0 | 7.1 | 0.90 |
| XII | XII Other and unspecified | 0 | 0 | 3 | 2 | 5 | 5 | 0.0 | 0.0 | 0.5 | 0.3 | 0.8 | 0.8 | 12 | 0.2 | 0.8 | 0.50 |
| | a Other specified | 0 | 0 | 1 | 0 | 1 | 3 | 0.0 | 0.0 | 0.2 | 0.0 | 0.2 | 0.5 | 4 | 0.1 | 0.3 | 0.25 |
| | b Unspecified | 0 | 0 | 2 | 2 | 4 | 2 | 0.0 | 0.0 | 0.3 | 0.3 | 0.6 | 0.3 | 8 | 0.2 | 0.5 | 0.67 |
| XIII | Langerhan's cell histiocytosis | 9 | 15 | 9 | 9 | 0 | 4 | 8.0 | 3.2 | 1.5 | 1.4 | 0.0 | 0.6 | 38 | 2.5 | 0.3 | 1.15 |
| | Males* ** | 103 | 432 | 328 | 317 | 528 | 695 | 177.9 | 180.4 | 103.8 | 97.3 | 167.1 | 219.6 | 3839 | 131.4 | 191.8 | – |
| | Females* ** | 86 | 378 | 229 | 226 | 374 | 620 | 156.3 | 166.4 | 76.5 | 73.4 | 121.0 | 198.8 | 3170 | 109.6 | 157.6 | – |
| | Total** | 189 | 810 | 557 | 543 | 902 | 1315 | 167.4 | 173.6 | 90.5 | 85.7 | 144.3 | 209.3 | 3511 | 120.8 | 174.9 | 1.25 |

* Gender specific incidence rates. ** Excludes Langerhan's cell histiocytosis. No cases of Kaposi's sarcoma (group IXc) were recorded. The cases classed as Burkitt's lymphoma were diagnosed exclusively after 1982 probably reflecting advances in histopathological classification hence this group is likely to be underrepresented in this series.

*© 2000 Cancer Research Campaign*

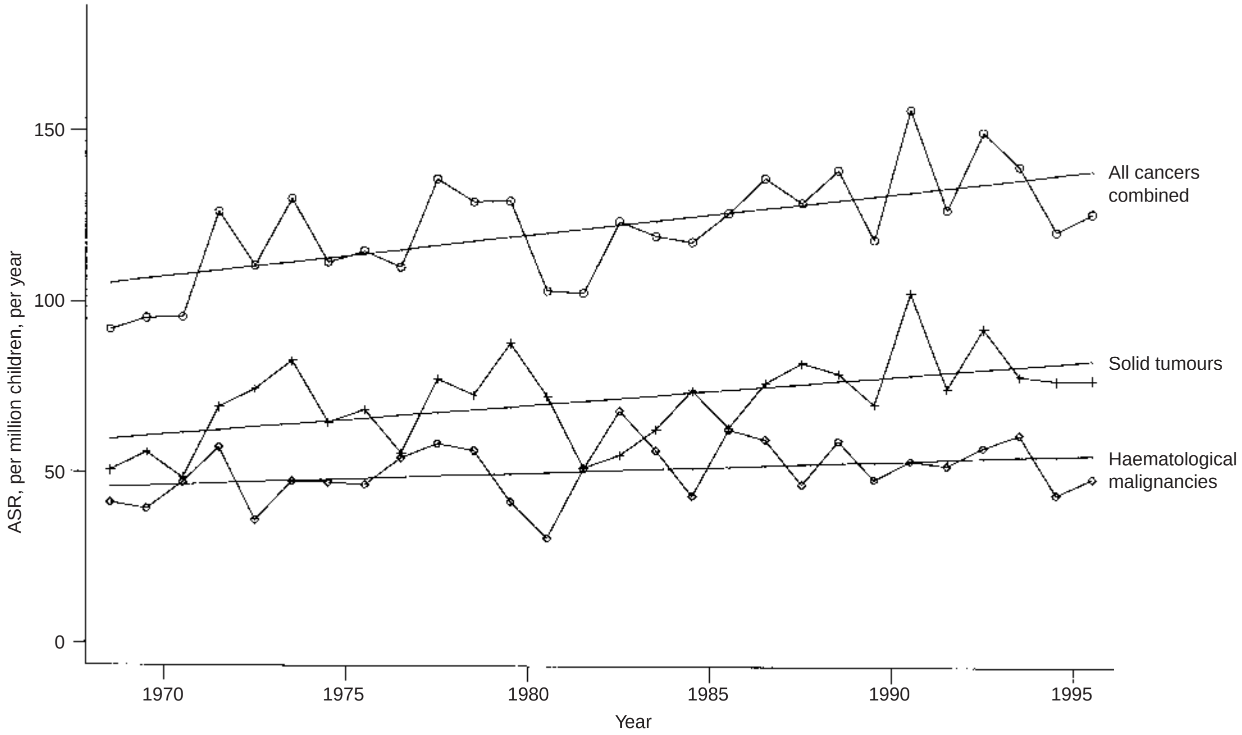

**Figure 2** Temporal changes in annual ASRs for the 0 to 14 year age-group by type of cancer.
Note: Data excludes LCH. Regression line shown for all cancers combined shows an increase in ASR by 12.2 per decade (95% CI 6.2 to 18.2). Solid tumours shows an increase in ASR by 8.6 per decade (95% CI 3.2 to 13.9). Haematological malignancies shows an increase in ASR by 3.6 per decade (95% CI-0.4 to 7.6)

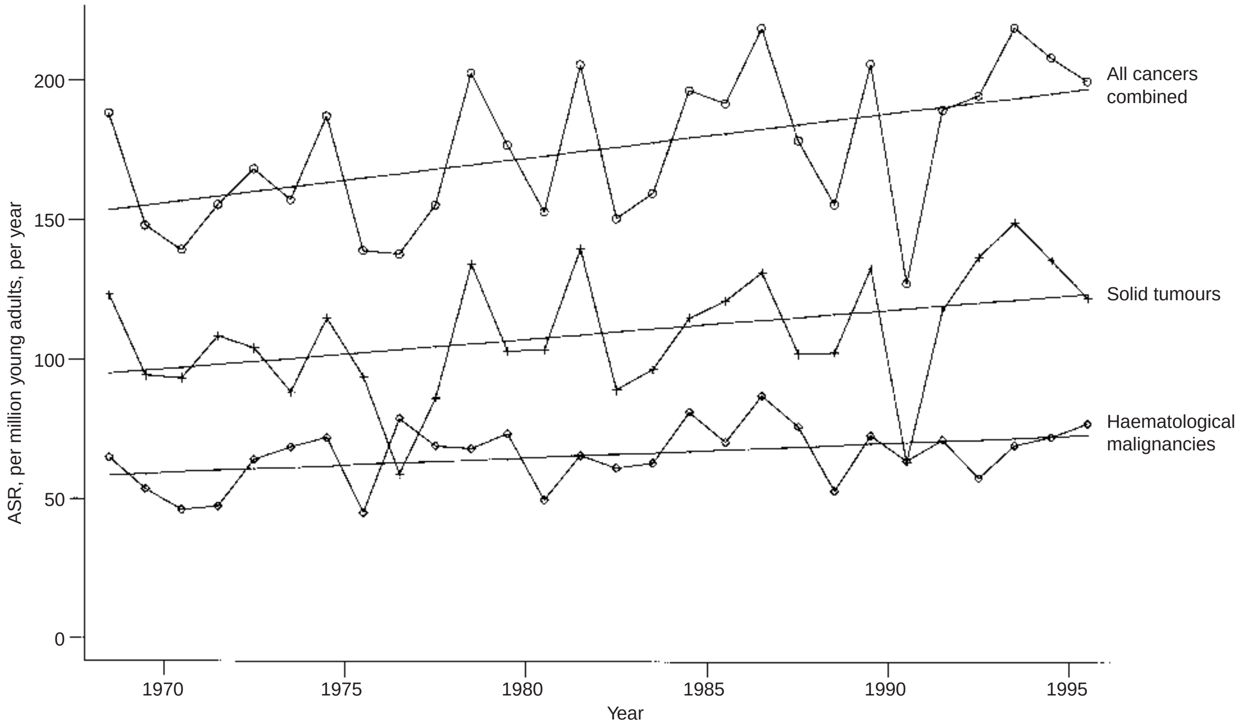

**Figure 3** Temporal changes in annual ASRs for the 15–24 age-group by type of cancer.
Note: Data excludes LCH. Regression line shown for all cancers combined shows an increase in ASR by 16 per decade (95% CI 5 to 27). Solid tumours shows an increase in ASR by 11 per decade (95% CI 1 to 20). Haematological malignancies shows an increase in ASR by 5 per decade (95% CI to 10)

*© 2000 Cancer Research Campaign*

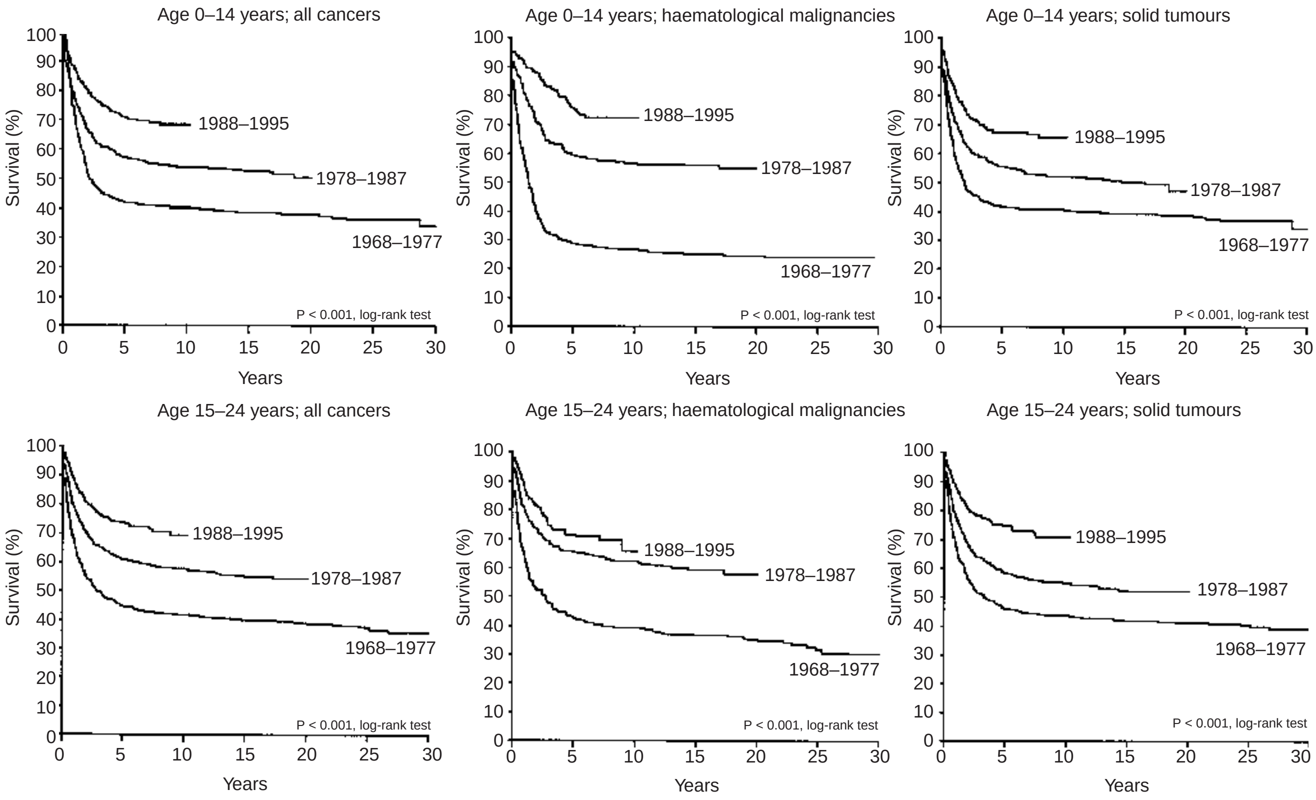

**Figure 4** Survival by time period for children and young adults

From 1968 to 1995 there were 32 million person years at risk for the 0 to 24 year age group. The age-standardized rates over the 28-year period for all cancers combined were 121 per million per year (95% confidence interval (CI) 116–126) for children, and 175 per million per year (95% CI 167–183) for young adults. Incidence rates (per million per year) varied by age-group; 167 for infants, 174 for 1–4 years, 91 for 5–9 years, 86 for 10–14 years, 144 for 15–19 years and 209 for 20–24 years. The cumulative incidence rate for cancer up to the age of 25 years was 3511 per million; i.e. a cumulative risk of 1 in 285. The ASR for childhood cancer in males was 131 per million children per year and 110 for females. For young adults (15–24 years) it was 192 for males and 158 for females.

There was a significant increase in the incidence of childhood cancers over the period (Figure 2); overall ASRs increased by 12 per million per decade ($P < 0.001$). Incidence of cancer in young adults increased over time (Figure 3) at a rate of 16 per million per decade ($P \leqq 0.008$).

### Follow-up

Median follow-up of the 2311 surviving patients was 12 years (range 1–31 years). For 2286 survivors the latest follow-up date was within 2 years of the date of analysis. Of those diagnosed prior to 1992 there were only eight with fewer than 5 years follow-up.

### Survival (Figure 4)

Five-year survival rates for children (0–14 years) over the time periods 1968–1977, 1978–1987, 1988–1995 were 42% (95% CI 38–45%), 57% (95% CI 54–61%) and 71% (95% CI 67–75%) respectively. For haematological malignancies 5-year survival rates over the three periods were 30% (95% CI 25–35%), 60% (95% CI 54–65%) and 77% (95% CI 71–82%) respectively. Survival rates for solid tumours were 41% (95% CI 37–46%), 56% (95% CI 51–60%) and 67% (95% CI 63–72%) respectively.

Five-year survival rates for young adults (15–24 years) over the three time periods 1968–1977, 1978–1987, 1988–1995 for all cancers were 45% (95% CI 42–49%), 62% (95% CI 58–65%) and 73% (95% CI 70–78%) respectively. For haematological malignancies 5-year survival rates over the three periods were 44% (95% CI 38–50%), 67% (95% CI 61–71%) and 72% (95% CI 66–78%) respectively. For solid tumours the rates were 46% (95% CI 41–51%), 58% (95% CI 54–62%) and 75% (95% CI 70–79%) respectively.

### DISCUSSION

Comprehensive cancer registration is a prerequisite for the effective planning of health services and evaluation of treatment efficacy. It also forms the basis for epidemiological studies which may lead to understanding of the aetiology of cancer. National registries give a broad picture of the situation but their data is

*© 2000 Cancer Research Campaign*

rarely as detailed or timely as is possible within a local registry. The NRYPMDR was one of the first population-based registries for childhood cancer and is the only registry in the UK having specialist information on the 15 to 24 year age group. It implements rigorous procedures to ensure that ascertainment is as complete as possible; cases are identified from multiple sources. Cancers in children have been registered prospectively from the onset of the registry in 1968 whilst the data on young adults were obtained retrospectively in 1985 in a very comprehensive exercise and have been collected prospectively since then. Small but significant increases in incidence over time are reported for both children and young adults. It is possible that there may have been under-ascertainment of young adults in the early period. However, the steady increase in incidence over time suggests a genuine increase in the underlying incidence of malignant disease (Figure 3).

### Childhood cancer

There were 2099 cases of cancer registered in children aged 0–14 years over the period 1968–1995 included in this study. Incidence rates for childhood cancer in the study region are generally similar to those reported by the UK National Registry of Childhood Cancers and to those in other Western populations (Parkin et al, 1988). The most common type of cancer was acute lymphoblastic leukaemia, accounting for 31% of cases with CNS tumours (23%) and forming the second largest group.

This report suggests a small but significant increase in the incidence of childhood cancer in the Northern Region, which is unlikely to be entirely accounted for by under-ascertainment in the early period: registration rates for paediatric cancers are considered to be in excess of 98%. The registry has consistently identified cases from multiple sources and treatment is centralized. Increased incidence is consistent with other population-based reports. Data from the USA indicated trends of increasing incidence of childhood cancer (Gurney et al, 1996), particularly in infants (Gurney et al, 1998; Kenney et al, 1998). There have been reports of increased incidence of specific tumours in the UK for neuroblastoma (Stiller, 1993) and leukaemia (Blair and Birch, 1994). The aetiology of childhood cancers remains largely unknown; only about 5% are thought to have an inherited genetic component (Narod et al, 1991). The increasing incidence of childhood cancer is consistent with the hypothesis that environmental factors play a role in the aetiology of these diseases. There is considerable evidence that the risk of childhood leukaemia in particular is influenced by characteristics of the environment, such as population mixing (Kinlen, 1995; Dickinson and Partier, 1999). For solid tumours the route by which the environment influences the risk of disease is unclear. The dramatic improvements in survival over the period mirror national and international trends in outcome (Stiller, 1994).

### Young adults

There is little published specifically concerning cancer incidence and survival in young adults, particularly in 20 to 24 year age group, with a few notable exceptions. This population-based study recorded 2217 cancers diagnosed in young adults. Lymphomas were the largest diagnostic group (26% of cases), with Hodgkin's disease being the most frequent diagnosis. Leukaemias accounted for 13% of cases and the proportions of acute myeloid and chronic myeloid types were higher than in children (38% and 9% vs 14% and 1% respectively).

There have been a small number of other population based reports including 15 to 19 year-olds. A study of cancer in Nordic countries (Tulinius et al, 1992) during 1981–1986 reported that incidence and distribution of cancer types in adolescents were similar to those seen in the North of England. In a study of adolescents (10–19 years) in New South Wales (Fritschi et al, 1995) over the period 1972–1991 incidence in the 15 to 19-year group was higher than in the North of England (rates per million per year: males 202 vs 167 and females 186 vs 121). This difference was largely due to the well known higher incidence of malignant melanoma which accounted for 27% of the Australian cases compared to 5% of cases in the North of England. A report from the SEER programme (Smith et al, 1999), for 1975–1994 also found higher overall incidence for 15- to 19-year-olds (rates 202 vs 144). Rates were similar for ALL (13 vs 13 per million, per year), AML (9 vs 9), Hodgkin's disease (33 vs 28), CNS (20 vs 23). However, SEER reported higher rates for germ-cell/gonadal (31 vs 13), thyroid cancer (15 vs 4), soft tissue sarcoma (16 vs 9), melanoma (14 vs 7) and NHL (15 vs 10). In common with the North of England the SEER data indicated a trend for increasing incidence of cancers in the 15 to 19 year-olds. Those cancers for which the differences in incidence rates were greatest included tumours which have been reported to be increasing in incidence (testicular cancer, melanoma and NHL) (Brown et al, 1987; Elder, 1995; Cartwright et al, 1999). Different racial characteristics of the populations may also affect the incidence of some types of cancer. Studies of 10- to 19-year-olds in Denmark (Martos et al, 1993) during 1943–1987 and Vaud in Switzerland (Levi et al, 1995) in 1974–1992 also found trends for increasing incidence of cancer in adolescents.

The diverse range of solid tumours in young adults may be categorized into three broad groups: (1) 'late' paediatric type cancers (groups IIIc, IV, VIa, VIIa, IXa), (2) 'age-specific' cancers for adolescents and young adults (groups VIII, Xc), and (3) 'early onset' carcinomas (group XI). The 'late' paediatric cancers normally associated with childhood included rhabdomyosarcoma (26 cases), medulloblastoma (14), neuroblastoma (seven), hepatoblastoma (two) and Wilms' tumour (one). There have been other anecdotal reports of such 'late' paediatric cancers in adult patients (Hollowood and Fletcher, 1994; Hentrich et al, 1995; Paterson and Walker, 1995; Moody et al, 1996; Varona and Ruiz, 1996). The cancers which are 'largely specific' to adolescents and young adults include bone tumours and testicular cancer. Bone tumours have a peak incidence between the ages of 10 and 20 years and accounted for 6% of cancers in young adults in this series; osteosarcoma and particularly Ewing's sarcoma are less common in patients older than 25 years (Dortman and Czerniak, 1995). Testicular cancer is most common in men in thier 20s; most of the men in this series had histological diagnoses of teratoma or seminoma while endodermal testicular cancer tended to predominate in boys aged under 15 years. There are many 'early onset' carcinomas arising in a wide range of anatomical sites. These include skin cancer (173 cases) with malignant melanoma being the most common type, thyroid carcinoma (59) and nasopharyngeal carcinoma (14). There are also carcinomas that are considered rare in young adults, particularly carcinomas of the breast (34), cervix (67), gastrointestinal system (43) and lung (16). The aetiology of these 'early onset' carcinomas remains largely unknown, though it is likely that there will be an inherited component.

© 2000 Cancer Research Campaign

Survival in the study region has improved considerably over the 28-year period, those diagnosed during 1988–1995 having a 5-year survival rate of 73% (95% CI 70–78). Improvements in survival in young adults have matched those seen in children and suggest that either many of the tumours of early adulthood are intrinsically more curable than those in older adults or that young adults receive more concentrated and effective treatment than is offered to many older adults, or a combination of the two. Those cancers with particularly good prognosis in this age-group include Hodgkin's disease, testicular cancer, thyroid carcinoma and non-melanoma skin cancer. For the cohort diagnosed between 1988 and 1995 the 5-year survival for Hodgkin's disease was 87%, (95% CI 80–93), testicular cancer 87% (95% CI 81–94), and at time of analysis there had been no deaths from thyroid carcinoma or non-melanoma skin cancers.

The number of survivors of cancer during childhood and early adulthood has increased and in this series 2088 survived at least 5 years. By the year 2025 approximately 1 in 400 of the adult population will be a survivor of cancer diagnosed before the age 25 years. The improvements in survival draw attention to the steadily growing population in whom study of psycho-social and other long-term effects for treatment of cancer is needed. Although there is considerable published and on-going research into late effects of treatment for childhood cancer, less is documented about late effects of treatment given to young adults. Specific studies of the late effects of diagnosis and treatment for cancer in young adults may be needed. The increase in incidence in the Northern region is cause for concern and requires further investigation.

### ACKNOWLEDGEMENTS

We thank all clinicians who so generously contributed their data to the registry. This analysis was supported by the North of England Children's Cancer Research Fund. The registry is funded by the Newcastle Hospitals NHS Trust. John Scott and John Wagget were the first two honorary secretaries of the registry.

### REFERENCES

Birch JM (1988) The Manchester Children's Tumour Registry 1954–1970 and 1971–1983. In: Parkin DM, Stiller CA, Draper GJ et al (eds) *International Incidence of Childhood Cancer* IARC Scientific Publications No 87, pp. 299–304. IARC, Lyon

Blair V and Birch JM (1994) Patterns and temporal trends in the incidence of malignant disease in children. I. Leukaemia and lymphoma. *Eur J Cancer* **30A**: 1490–1498

Brown LM, Pottern LM and Hoover RN (1987) Testicular cancer in young men: the search for causes of the epidemic increase in the United States. *J Epidemiol Comm Health* **41**: 349–354

Cartwright R, Brincker H and Carli PM et al (1999) The rise in incidence of lymphomas in Europe 1985–1992. *Eur J Cancer* **35**: 627–633

Compton E (1972) *Local Government Boundary Commission Reports.* HMSO, London

Craft AW, Amineddine HA, Scott JE and Wagget J (1987) The Northern Region Children's Malignant Disease Registry 1968–1982: incidence and survival. *Br J Cancer* **56**: 853–858

Craft AW, Parker L, Openshaw S, et al (1993) Cancer in young people in the North of England, 1968–85: analysis by census wards. *J Epidemiol Community Health* **47**:

Day NE (1992) Cumulative rate and cumulative risk. In: Parkin DM, Muir CS, Whelan SL, Gao YT, Ferlay J and Powel J (eds) pp. 862–864. *Cancer Incidence in Five Continents VI.* IARC Scientific Publications, Lyon

Dickinson HO and Parker L (1999) Quantifying the effect of population mixing on childhood leukaemia risk: the Seascale cluster. *Br J Cancer* **81**: 144–151

Dorfman HD and Czerniak B (1995) Bone cancers. *Cancer* **75**: 203–210

Elder DE (1995) Skin cancer: melanoma and other specific non-melanoma skin cancers. *Cancer* **S75**: 245–256

Fritschi L, Coates M and McCredie M (1995) Incidence of cancer among New South Wales adolescents: which classification scheme describes adolescent cancers better? *Int J Cancer* **60**: 355–360

Gurney JG, Davis S and Severson RK et al (1996) Trends in cancer incidence among children in the US. *Cancer* **78**: 532–541

Gurney JG, Ross JA and Wall DA et al (1997) Infant cancer in the US: histology-specific incidence and trends, 1973 to 1992. *J Pediatr Haematol/Oncol* **19**: 428–432

Hentrich MU, Meister P and Brack NG et al (1995) Adult Wilms' tumour. Report of two cases and a review of the literature. *Cancer* **75**: 545–551

HMSO (1996) *Regional Trends 31*. HMSO, London, pp. 203–204

Hollowood K and Fletcher CD (1994) Rhabdomyosarcoma in adults. *Semin Diagn Pathol* **11**: 47–57

Kaplan EL and Meier P (1958) Non-parametric estimation from incomplete observations. *J Am Stat Assoc* **53**: 457–481

Kenney LB, Miller BA and Gloeckler Ries LA et al (1998) Increased incidence of cancer in infants in the US: 1980–1990. *Cancer* **82**: 1396–1400

Kinlen LJ (1995) Epidemiological evidence for an infective basis in childhood leukaemia. *Br J Cancer* **71**: 1–5

Kramarova E, Stiller CA, Ferlay J et al (1996) *International Classification of Childhood Cancer.* IARC Technical Report. IARC, Lyon

Levi F, La Vecchia C, Randimbison L and Te VC (1995) Cancer incidence and mortality among teenagers in Vaud, Switzerland, 1974–1992. *Int J Cancer* **61**: 40–43

Martos MC, Winther JF and Olsen JH (1993) Cancer among teenagers in Denmark, 1943–1987. *Int J Cancer* **55**: 57–62

Moody AM, Norman AR and Tait D (1996) Paediatric tumours in the adult population: the experience of the Royal Marsden Hospital 1974–1990. *Med Pediatr Oncol* **26**: 153–159

Narod SA, Stiller CA and Lenoir GM (1991) An estimate of the heritable fraction of childhood cancer. *Br J Cancer* **63**: 993–999

Parkin DM, Stiller CA, Draper et al (1988) *International Incidence of Childhood Cancer.* IARC Scientific Publications No 87. IARC, Lyon

Paterson K and Walker RW (1995) Meduloblastoma/primitive neuroectodermal tumour in 45 adults. *Neurology* **45**: 440–442

Smith PG (1992) Comparison between registries: age-standardized rates. In Parkin DM, Muir CS, Whelan SL, Gao YT, Ferlay J and Powel J (eds), pp. 865–870. *Cancer Incidence in Five Continents VI.* IARC Scientific Publications, Lyon

Smith MA, Gurney JG and Gloeckler Ries LA (1999) Cancer among adolescents 15–19 years old. In: SEER Pediatric Monograph, The National Cancer Institute, Bethesda

Stiller CA (1993) Trends in neuroblastoma in Great Britain: incidence and mortality, 1971–1990. *Eur J Cancer* **29A**: 1008–1012

Stiller CA (1994) Population based survival rates for childhood cancer in Britain, 1980–1991. *Br Med J* **309**: 1612–1616

Tulinius H, Storm HH and Pukkala E et al (1992) Cancer in the Nordic countries 1981–86. *APMIS* **100**: 126–133

Varona L and Ruiz J (1996) Adult-onset neuroblastoma with intraspinal (dumbbell) extension. *Neurologia* **11**: 88–89

WHO (1990) *International Classification of Diseases for Oncology* 2nd edn. WHO, Geneva

© 2000 Cancer Research Campaign
